# The effect of heat mitigation strategies on thermoregulation and productivity during simulated occupational work in the heat in physically active young men

**DOI:** 10.3389/fspor.2023.1274141

**Published:** 2024-01-11

**Authors:** Margaret C. Morrissey-Basler, Gabrielle J. Brewer, Travis Anderson, William M. Adams, John S. Navarro, Monique Marcelino, David G. Martin, Douglas J. Casa

**Affiliations:** ^1^ Department of Kinesiology, Korey Stringer Institute, University of Connecticut, Storrs, CT, United States; ^2^ Department of Health Sciences, Providence College, Providence, RI, United States; ^3^ Department of Sports Medicine, United States Olympic & Paralympic Committee, Colorado Springs, CO, United States; ^4^ United States Coalition for the Prevention of Illness and Injury in Sport, Colorado Springs, CO, United States; ^5^ School of Sport, Exercise and Health Sciences, Loughborough University, Leicestershire, United Kingdom; ^6^ Department of Kinesiology, University of North Carolina at Greensboro, Greensboro, NC, United States

**Keywords:** body cooling, hydration, occupational, heat stress, prevention

## Abstract

**Purpose:**

To investigate heat stress mitigation strategies on productivity and thermoregulatory responses during simulated occupational work in the heat.

**Methods:**

Thirteen physically active men (age, 25 ± 4 years; body mass,77.8 ± 14.7 kg; VO_2_peak, 44.5 ± 9.2 ml·kg^−1^·min^−1^) completed five randomized-controlled trials in a hot environment (40°C, 40% relative humidity). Each trial was 4.5 h in duration to simulate an outdoor occupational shift. Thermoregulatory responses (heart rate, HR; rectal temperature, Trec; mean skin temperature, Tsk), perceptual responses (rating of perceived exertion, RPE; thermal sensation; thermal comfort; fatigue) and productivity outcomes (box lifting repetitions, time to exhaustion) were examined in the following heat mitigation strategy interventions: (1) simulated solar radiation with limited fluid intake [SUN]; (2) simulated solar radiation with no fluid restrictions [SUN + H2O]; (3) shade (no simulated solar radiation during trial) with no fluid restrictions [SHADE + H_2_O]; (4) shade and cooling towels during rest breaks with no fluid restrictions [COOL + H_2_O]; and (5) shade with cooling towels, cooling vest during activity with no fluid restrictions [COOL + VEST + H_2_O].

**Results:**

[COOL + VEST + H_2_O] had lower Trec compared to [SUN] [*p* = 0.004, effect size(ES) = 1.48], [SUN + H_2_O] (*p* < 0.001, ES = −1.87), and [SHADE + H_2_O] (*p* = 0.001, ES = 1.62). Average Tsk was lower during the treadmill and box lifting activities in the [COOL + VEST + H_2_O] compared to [SUN] (*p* < 0.001, ES = 7.92), [SUN + H_2_O] (*p* < 0.001,7.96), [SHADE + H_2_O] (*p* < 0.001), and [COOL + H_2_O] (*p* < 0.001, ES = 3.01). There were performance differences during the [COOL + VEST + H_2_O] (p = 0.033) and [COOL + H_2_O] (*p* = 0.023) conditions compared to [SUN] during phases of the experimental trial, however, there were no differences in total box lifting repetitions between trials (*p* > 0.05).

**Conclusion:**

Our results suggest that during a simulated occupational shift in a laboratory setting, additional heat mitigation strategies ([COOL + VEST + H_2_O] and [COOL + H_2_O]) reduced physiological strain and improved box lifting performance to a greater degree than [SUN]. These differences may have been attributed to a larger core to skin temperature gradient or reduction in fatigue, thermal sensation, and RPE during [COOL + H_2_O] and [COOL + VEST + H_2_O]. These data suggest that body cooling, hydration, and “shade” (removal of simulated radiant heat) as heat stress mitigation strategies should be considered as it reduces physiological strain while producing no additional harm.

## Introduction

With the ever increasing surface and sea temperatures as a result of climate change, the global economic losses associated with occupational heat stress are predicted to be at least 2.4 trillion U.S dollars by 2030 (1). This negative economic impact is primarily driven by both increased heat-related injuries and reduction in work capacity or “productivity” (1–5). Current research has focused on quantifying the influence of environmental heat stress on productivity to motivate employers to make changes to work conditions to reduce heat-related events (4–9). Foster et al. (2021) reported that while using an advanced empirical model to estimate reductions in work capacity as a function of wet bulb globe temperature (WBGT), mild heat stress (WBGT = 18°C) can result in a 10% reduction in physical work capacity and extreme heat stress (WBGT = 40°C) can result in 78% reduction in physical work capacity (10).

To alleviate the effect of occupational heat stress on productivity, heat stress mitigation strategies have been proposed as a solution. Although many employers perceive heat safety interventions such as heat acclimatization, work-to-rest ratios, and body cooling to impede economic growth, it must be recognized that these interventions can result in enhanced productivity outcomes (2, 8, 9). For example, Morabito et al. (2021) reported that moving a work shift 2 h earlier to limit heat exposure reduced overall cost by 33% (8). As work rate has been shown to influence thermal strain, many employers have begun to emphasize “self-pacing” as a protective strategy against heat stress (11). Research studies have suggested that *informed* workers can be encouraged to self-pace and regulate their workload in hot conditions to alleviate extreme physiological strain (11, 12). Although this strategy is appealing, industries such as agriculture and construction that have the most reported heat-related illnesses in the United States (13) may not permit this to occur given these industries pay structures (e.g., piece pay in agriculture) or contractual obligations (e.g., construction site deadlines and deliverables).

Heat mitigation strategies such as increased fluid accessibility and body cooling are interventions that often do not disrupt everyday working procedures and have been shown to protect against heat-related illnesses (14–17). Implementing body cooling and hydration strategies can increase thermal comfort (18, 19), reduce core temperature (14, 15), and enhance productivity (20–22). Existing literature in sport settings has reported that body cooling during physical activity improves performance in the heat by 4%–19% (15, 20). However, there are no studies that have quantified the influence of heat mitigation strategies on thermoregulation and productivity during simulated occupational work in the heat. Therefore, the aim of the current study was to evaluate the influence of different heat mitigation strategies on thermoregulatory responses and productivity during simulated occupational work in a hot environment. It was hypothesized that additive heat mitigation strategies (e.g., shade, increased fluid accessibility, and body cooling during physical activity and rest) would result in lower thermoregulatory strain and improved productivity when compared to simulated solar radiation condition with limited fluid accessibility.

## Methods

### Participants

Thirteen physically active, healthy men (age, 25 ± 4 years; body mass, 77.8 ± 14.7 kg; VO_2_peak, 44.5 ± 9.2 ml·kg^−1^·min^−1^) volunteered to participate in this study. Participants were eligible if they were between 18 and 45 years old and had no reported chronic disease, musculoskeletal injuries, contraindications to sun exposure, or taking medications that influence body temperature (i.e., amphetamines, antihypertensives, anticholinergics, acetaminophen, diuretics, aspirin). All study procedures and potential risks were explained to participants verbally prior to obtaining written and informed consent. All study procedures were approved by the Institutional Review Board at University of Connecticut.

### Study design

**Figure 1:**
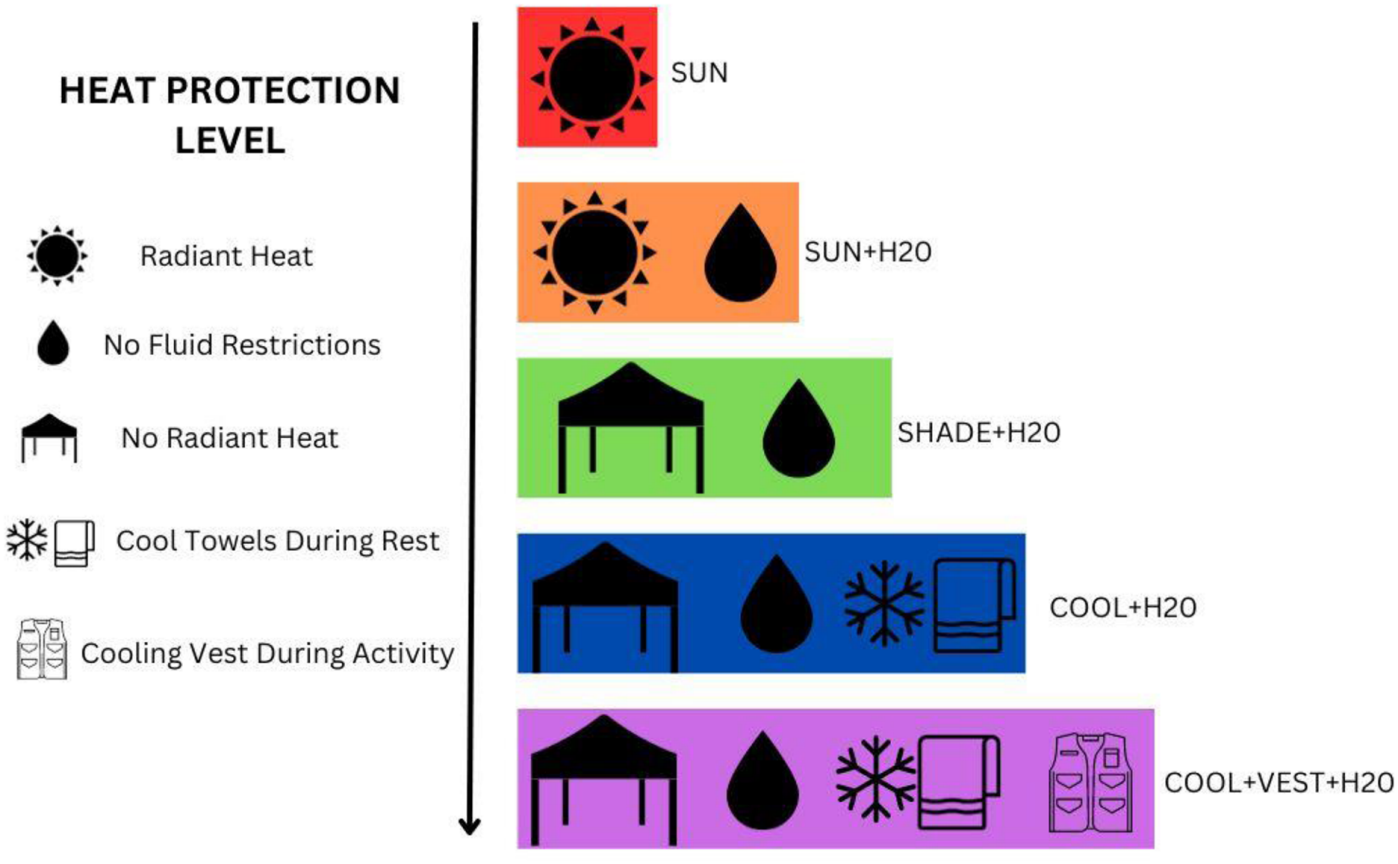
Experimental trials.

This study implemented a randomized, cross-over design consisting of eight study visits: one baseline visit, two familiarization trials, and five experimental trials. The order in which the participants performed each experimental trial was randomized by selecting from a pool of all possible ordered scenarios. Of the 120 possible combinations that were randomly generated, the first 13 were selected. For the five experimental trials, physiological responses and productivity outcomes were measured during simulated occupational work under five heat stress mitigation scenarios: (1) radiant heat exposure during activity and rest with *ad libitum* fluid intake during rest only [SUN]; (2) radiant heat exposure during activity and rest with *ad libitum* fluid intake permitted during both activity and rest [SUN + H_2_O]; (3) no radiant heat exposure during exercise or rest and *ad libitum* fluid intake during activity and rest [SHADE + H_2_O]; (4) no radiant heat exposure during exercise or rest, cooling towels during rest, and *ad libitum* fluid intake during rest and exercise [COOL + H_2_O]; and (5) no radiant heat exposure during exercise or rest, cooling vests during activity, and cooling towels during rest, and *ad libitum* fluid intake during activity and rest [COOL + VEST + H_2_O] (Figure 1). For all experimental trials, participants donned long pant overalls, long sleeve shirt, gloves, and a baseball cap to reflect the personal equipment clothing worn by outdoor occupational workers. The clothing was selected to mimic clothing worn by construction or agriculture workers since these working populations report the highest rates of heat illness (13).

### Baseline and familiarization visits (visits 1–3)

All baseline and familiarization visits were performed in temperate conditions (∼22°C). For the baseline visit, participants reported to the laboratory following a 3 h fast. Upon arrival, a urine sample was collected to record hydration status (urine specific gravity; Atago Co, Tokyo, Japan, model 300 CL) and nude body mass was measured on a physician beam scale (Defender R7000 Extreme; OHAUS Corp). Body composition was assessed using air displacement plethysmography (BOD POD, Cosmed; Rome, Italy). After anthropometric assessments, participants performed a peak oxygen consumption test (VO_2_peak) on a treadmill to assess cardiorespiratory fitness and prescribe exercise intensity for the experimental trials. The VO_2_peak test consisted of two min stages that progressively increased in speed (by 0.5–1.0 mph increments) until the participant reached volitional exhaustion. Heart rate (HR; Polar® H10, Polar, Inc., Kempele, Finland) and rating of perceived exertion were collected at the end of each stage. VO_2_ and RER were collected continuously (30-sec average) using a Hans Rudolph metabolic mask (Hans Rudolph Inc., Shawnee, KS, USA) attached to a hose and delivered to the metabolic cart system (Parvo Medics Truemax 2400 Metabolic Measurement System, Consentius Technologies, Sandy, UT). Volitional exhaustion was characterized as the criterion to reach VO_2_peak.

Following the VO_2_peak test, participants were familiarized with the box lifting activity (see box lifting activity section), and the time to exhaustion test. Participants were required to re-visit the laboratory for two more familiarization trials of the box lifting activity. During the familiarization visits, participants performed 1–2 repetitions of box lifting activity with coaching from the research staff. No other measures were collected during the familiarization visits.

### Experimental trials (visits 4–8)

Participants abstained from caffeine and alcohol prior to each experimental visit for 12 and 24 h, respectively. Participants were asked to arrive in a hydrated and fed state at the same time of day (∼11:30am) and provided a urine sample to ensure that they were normally hydrated (urine specific gravity <1.020) Participants provided a measure of their nude body mass and then were asked to self-insert a rectal thermistor 10 cm past the anal sphincter. Participants donned a HR monitor and four skin temperature (Tsk) sensors were affixed to their chest, arm, thigh, and calf (Maxim Integrated; San Jose, CA) for assessment of mean Tsk. Mean Tsk was calculated using the following equation from Ramanathan (23):

MeanTsk=(0.3×Tchest)+(0.3×Tarm)+(0.2×Tthigh)+(0.2×Tcalf)

Where mean Tsk = mean skin temperature, Tchest = chest skin temperature, Tarm = arm skin temperature, Tthigh = thigh skin temperature, Tcalf = calf skin temperature.

HR, rectal temperature (Trec), and Tsk were collected every minute throughout the protocol. The core to skin gradient was calculated using both Trec and Tsk. After instrumentation of all study equipment, participants donned the assigned study ensemble (long pant overalls, a long sleeve shirt, gloves, and a baseball cap).

### Experimental protocol

**Figure 2:**
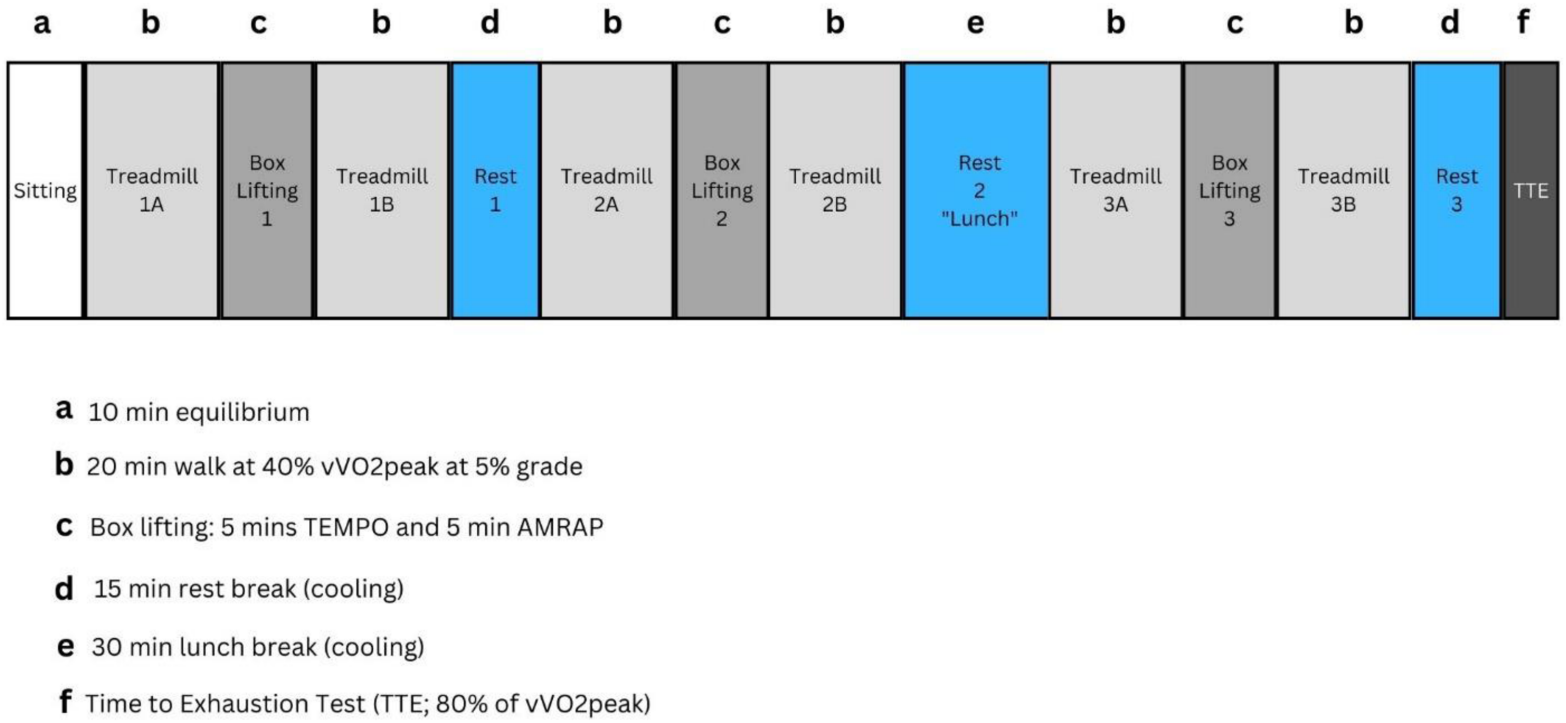
Experimental trial protocol. vVO2peak, velocity at peak oxygen consumption.

An overview of the experimental protocol is presented in Figure 2. Participants entered the environmental chamber (40°C, 40% relative humidity; Cantrol Environmental Systems, ON, Canada) and were seated for 10 min prior to starting all physical activity procedures. The environmental conditions of 40°C, 40% relative humidity were selected to simulate extreme heatwave effects that occur in the southwest and south regions of the U.S. The participants performed 3 cycles of the following activity and rest protocol: a 20 min treadmill walk, 10 min box lifting activity, 20 min treadmill walk, and a rest break. Cycles 1 and 3 included a 15 min rest break, and Cycle 2 included an extended rest break (30 min) to simulate a “lunch break”. Each treadmill walk was performed at a 5% grade at 30% of the participants’ velocity at VO_2_peak(vVO_2_peak). Participants were asked to report their rating of perceived exertion, thermal sensation, thermal comfort, and fatigue (perceptual data) at the end of each activity. With the exception of [SUN], participants drank ad libitium throughout the entire experimental protocol. Participants were able to drink ad libitium during all rest breaks but were not permitted to drink during any other part of the trial. Participants ended the trial with a time to exhaustion test on the treadmill. For the time to exhaustion test, participants were required to run at 80% of their vVO_2_peak until they reached volitional exhaustion. Participants were prohibited from completing the time to exhaustion task if their Trec was over 39.75°C.

### Measures

#### Box lifting activity

**Figure 3:**
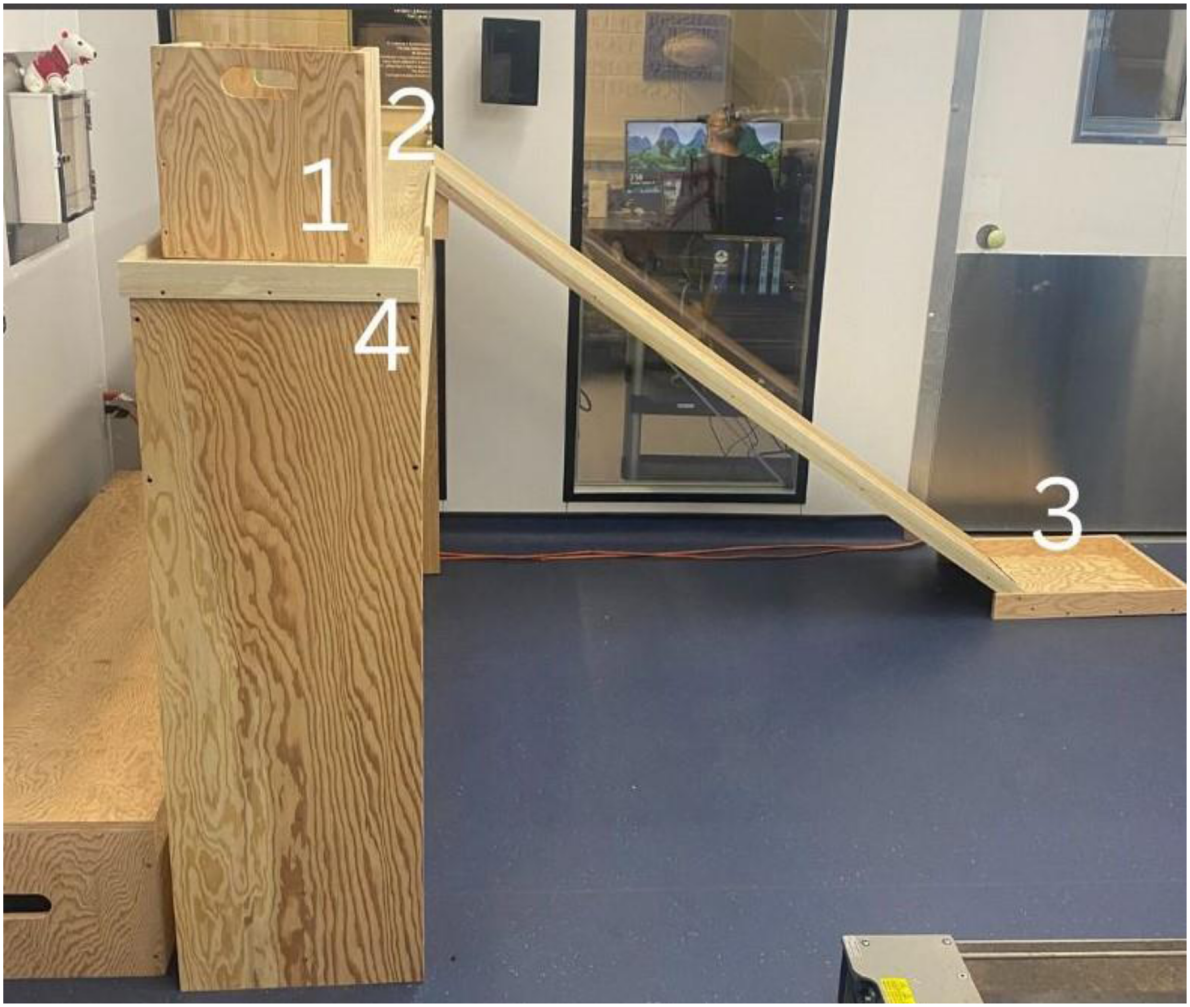
Box lifting Set up and protocol instructions. 1 = starting point; 2 = point 2, where participants slid the box from 1 to 2; 3 = point 3, participants slid the box down the slide from point 2 to point 3; 4 = point 4, participants bent down and picked up the box with both hands and moved it back to the starting point.

The box lifting activity consisted of participants repetitively moving and lifting boxes within a box lifting apparatus. The box lifting apparatus was 4 feet tall, 6 feet wide, and the slide was 5.6 feet long. The box weight was 7% of the participant's total fat-free body mass (mean box weight: 4.62 ± 0.70 kg). A percentage of total fat-free body mass was selected to standardize the weight and account for cardiorespiratory fitness differences (24). From the starting point of the box lifting activity (Figure 3) on an elongated shelf (top left side), the participant slid the box horizontally to the right until it reached the top of the slide. They then held the top of the box as it slid down the slide until it reached the stopper at the bottom. Participants were then required to bend down and pick up the box with both hands (legs straddling the box, participant bending their knees). The participant walked the box over to the start of the box lifting activity and lifted it onto the elongated shelf. The movement from start to finish through the box lifting activity was counted as one repetition.

Each cycle of the box lifting activity was broken into two parts: (1) the “TEMPO” phase and (2) the As Many Rounds as Possible “AMRAP” phase. There was one minute of rest between phases. The 5 min “TEMPO” phase consisted of the participant moving the box through the activity based on a metronome. The “TEMPO” phase was designed to simulate conditions where self-pacing during occupational work is not available. The metronome was set at 20 bpm and participants were asked to move from point 1 to 2 within the metronome beats (Figure 3) and were asked to follow the same protocol between points 2 to 3, and 3 to 4. The second phase, the 5 min “AMRAP” phase, was designed to simulate self-paced working conditions where participants moved through the box lifting activity as quickly as possible during the 5 min phase. In both the “TEMPO” and “AMRAP” phases, the number of repetitions were counted and were representative of a worker's productivity in self-pacing and non-self-pacing scenarios. The total number of boxes lifted per phase within each cycle and the total number of boxes lifted in the experimental trial day were calculated as a productivity metric. The TEMPO phase was only included in the total number of boxes lifted calculation and not per cycle. The number of repetitions were included as not all participants could maintain the pace of the metronome during the activity.

#### Simulated solar radiation

For the [SUN] and [SUN + H_2_O] trials, simulated solar radiation was performed using radiant heat lamps (Super PAR CP60 EXC 240 V 1,000 W lamps). Research staff utilized a potentiometer to regulate the intensity of solar radiation and ensure the solar radiant level was set at 800 W/m^2^. This is classified as a typical level of solar radiation during work under clear sky conditions. Participants were required to wear sunscreen and ultraviolet sunglasses during these trials. Simulated solar radiation was only present in the [SUN] and [SUN + H_2_O] trials.

#### Body cooling

**Figure 4:**
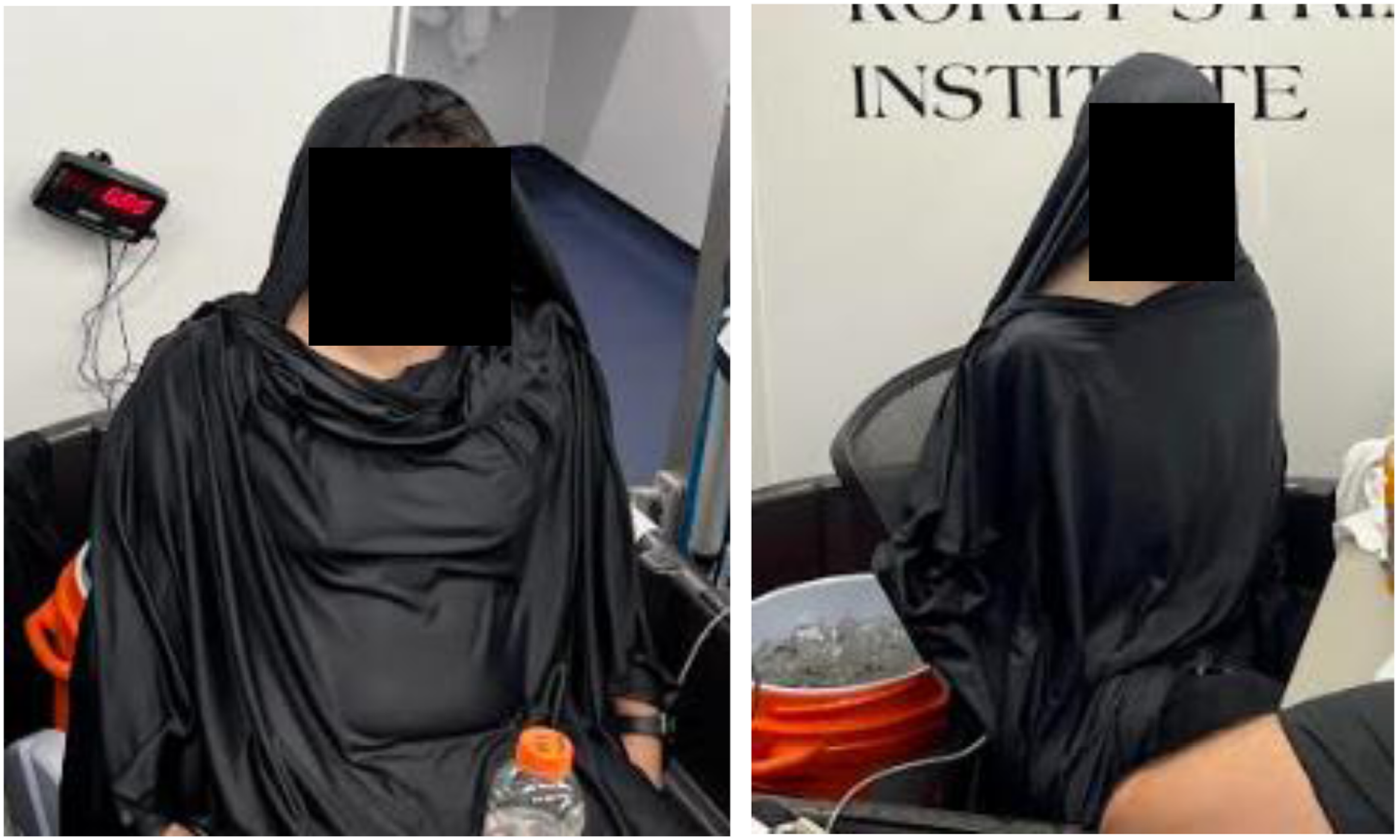
Towel placement during body cooling during rest breaks.

For [SHADE + H_2_O], [COOL + H_2_O], and [COOL + VEST + H_2_O], participants took a seated position during rest breaks. For [COOL + H_2_O] and [COOL + VEST + H_2_O], participants were wrapped in two large cooling towels (MISSION, MPUSA, LLC, Hawthorne, New York, USA) during all three rest breaks. Participants removed their hat, gloves, overalls, and shirt prior to initiating cooling. The two cooling towels were placed on the participant to cover as much body surface area as possible. One towel draped over the participants shoulders, head, and arms and the other was placed over their chest and thighs (Figure 4). The towels were submerged in cold water (∼2.0°C) prior to placing on the participant. The towels were rotated every 5 min to maximize cooling capacity during the rest period. The [COOL + VEST + H_2_O] trial included a cooling vest (Oro Sports USA, Buffalo, New York, USA) worn during the equilibrium rest period, treadmill walking, and box lifting activities. The cool packs from the cooling vest were replaced every 30 min.

#### Lunch break

Cycle 2 included a 30 min rest break that simulated a “lunch break”. Participants were provided peanut butter and jelly sandwiches, potato chips, and a carbohydrate sports drink (Gatorade Thirst Quencher). On average, participants consumed approximately 701 kcals of peanut butter and jelly sandwiches, 172 kcals of chips, and 152 kcals of carbohydrate sport drink at each trial calculated based on the volume of each food consumed. Food was not standardized as the caloric needs of individuals vary and are not standardized in a work environment.

#### Statistical approach

To our knowledge, there were no direct data on the effect of different heat stress mitigation strategies on physiological responses or productivity during a simulated occupational work environment. Therefore, we used previous published Trec data from Butts et al. (25) that examined the physiological effects of a cooling garment during simulated industrial work in the heat to calculate an *a priori* power calculation (G-Power version 3.1.9.7). The power calculation determined that 12 participants were required to achieve 80% of power with an alpha of 0.05. Thirteen participants were recruited for this study to account for attrition rates and therefore, all thirteen participants’ data were used in this study.

Linear mixed-effects models were used to model differences in study conditions. After fitting the model, estimated marginal means with Tukey post-hoc corrections were used to make statistical inferences between study conditions, and Cohen's d effect sizes were calculated for each pairwise comparison. Models comparing box lift repetitions included a fixed effect for condition and its interaction with box lift rounds and perceptual data (RPE, thermal, fatigue, comfort) were modeled as three-way interactions between time (pre vs. post), treadmill task (1A, 1B, 2A, etc.), and condition. The same model specification was used to compare Trec and Tsk between conditions for box lifting and treadmill tests, however, temperature models also covaried for the mean HR response under each event and testing condition to control for potential differences in participant effort and intensity between conditions. All models included a random intercept for participants to account for inter-individual variability. Alpha level was set at *p* < 0.05 for all comparisons, and Cohen's d was interpreted as a trivial (Cohen's *d* < 0.2), small (Cohen's *d* = 0.2–0.5), medium (Cohen's *d* = 0.5–0.8), or large (Cohen's *d* > 0.8) effect. Effect sizes (ES) are presented with 95% confidence intervals (95% C.I.). All statistical analyses were completed in R Statistical Software (26) and utilized the *lme4* (27) and *emmeans* (28) packages. Data are reported as trial-level comparisons (mean centered values), and cycle level comparisons, respectively. Trec and Tsk are reported as overall trial comparisons, trial comparisons in rest, treadmill, and box lifting periods, and cycle level comparisons, respectively.

## Results

### Participant characteristics

Participant characteristics are presented in Table 1.

**Table 1:** Participant characteristics (*N* = 13).

	Mean (SD)	Range
Age (years)	25 ± 4	19–34
Height (cm)	173.8 ± 10.9	155.5–191.0
Body mass (kg)	77.8 ± 14.7	51.8–104.1
Body fat (%)	15.1 ± 7.0	3.8–26.2
VO2peak (ml·kg^−1^·min^−1)^	44.5 ± 9.2	33.1–69
vVO2peak (mph)	14.8 ± 2.1	11.–18.5

VO2peak, peak oxygen consumption; vVO2peak velocity at peakoxygen consumption;Cm, centimeter; kg, kilogram; %, percent; ml·kg^−1^·min^−1;^ milliliters per kilogram per minute; mph, miles per hour.

### Rectal temperature

For overall trial level comparisons, [COOL + VEST + H_2_O] had significantly lower Trec compared to [SUN] (*p* = 0.004, ES = 1.48, large effect, 95% C.I = 0.631, 2.329), [SUN + H_2_O] (*p* < 0.001, ES = 1.87, large effect, 95% C.I = 0.996, 2.748), and [SHADE + H_2_O] (*p* = 0.001, ES = 1.62, large effect, 95% C.I = 0.766, 2.483). There were no differences in baseline rectal temperature between the days they performed each trial (i.e., no acclimatization effect, *p* = 0.897; Supplementary Table S1).

Table 2 presents overall Trec data across all three cycles during rest, box lifting and treadmill periods. The mean difference in Trec between minimum and maximum Trec during the treadmill activity across all three cycles was 1.42°C, 1.60°C, 1.37°C, 1.11°C, and 0.98°C in the [SUN], [SUN + H_2_O], [SHADE + H_2_O], [COOL + H_2_O], and [COOL + VEST + H_2_O], conditions, respectively (Table 2). These differences were statistically significant between the [COOL + VEST + H_2_O] compared to the [SUN] (*p* = 0.004, ES = 1.400, *large effect*, 95% CI = 0.601–2.199), [SUN + H_2_O] (*p* < 0.001, ES = 1.96, *large effect*, 95% C.I = 1.147–2.772), and the [SHADE + H2O] (*p* = 0.018, ES = 1.223, *large effect*, 95% C.I = 0.428–2.019), but not [COOL + H2O] (*p* = 0.820, ES = 0.421, *small effect*, 95% CI = −0.364–1.206) conditions. During the treadmill and box lifting activity across all three cycles, maximum Trec was significantly lower in [COOL + H_2_O] condition compared to the [SUN + H_2_O] (treadmill: *p* < 0.001, ES = 1.768, *large effect*, 95% CI = 0.941–2.595, box lifting: *p* = 0.040, ES = 1.244, *large effect*, 95% CI = 0.343–2.146), and lower in [COOL + VEST + H_2_O] compared to [SUN] (treadmill: *p* < 0.001, ES = 1.767, *large effect*, 95% CI = 0.941–2.594, box lifting: *p* = 0.006-, ES = 1.416, *large effect*, 95% CI = 0.565–2.267), [SUN + H2O] (treadmill: *p* < 0.001, ES = 2.646, large effect, 95% CI = 1.791–3.501, box lifting: *p* < 0.001, ES = 1.826, *large effect*, 95% CI = 0.926–2.726), and [SHADE + H2O] (treadmill: *p* < 0.001, ES = 1.912, *large effect*, 95% CI = 0.069–1.687, box lifting: *p* = 0.015, ES = 1.331, *large effect*, 95% CI = 0.464–2.197). During rest, there were no statistically significant differences in cooling rate between trials (*p* > 0.05).

**Table 2:** Mean descriptive statistics for rectal and skin temperature during rest, Box lifting, and treadmill period.

	Rest					Box lifting					Treadmill				
Trial	Min	Average	Max	Diff	Rate (C·min^−1^)	Min	Average	Max	Diff	Rate (C·min^−1^)	Min	Average	Max	Diff	Rate (C·min^−1)^
Rectal temperature															
SUN	38.01	38.44	38.87	0.87	−0.0122	37.87	38.3	38.61	0.73	0.0168	37.43	38.26	38.85	1.42	0.0112
SUN + H_2_O	38.17	38.6	38.99	0.82	−0.0139	37.86	38.32	38.73	0.86	0.0157	37.54	38.37	39.14	1.6	0.0117
SHADE + H_2_O	38.02	38.48	38.89	0.88	−0.0186	37.79	38.22	38.53	0.74	0.0148	37.53	38.3	38.9	1.37	0.0111
COOL + H_2_O	37.84	38.17^b^	38.52^b^^c^	0.68	−0.015	37.64	37.98	38.28^b^	0.64	0.0098	37.45	38.08	38.56^b^	1.11^b^	0.0093
COOL + VEST + H_2_O	37.48^a^^b^^c^	37.9^a^^b^^c^	38.28^a^^b^^c^	0.8	−0.0179	37.57	37.86^a^^b^^c^	38.12^a^^b^^c^	0.54	0.0085	37.29	37.86^a^^b^^c^	38.27^a^^b^^c^	0.98^a^^b^^c^	0.0104
Skin temperature															
SUN	36.27	37.13	37.89	1.62	−0.0006	36.12	37.03	37.8	1.68	−0.0322	35.52	36.92	38.06	2.54	0.0217
SUN + H_2_O	36.47	37.35	38.25	1.78	0.0054	36.13	37.11	37.81	1.68	−0.0106	35.42	37.04	38.37	2.94	0.0193
SHADE + H_2_O	36.2	36.8	37.46	1.26	0.0064	36.08	36.69	37.14	1.07	−0.0003	34.74	36.59	37.4^b^	2.65	0.0143
COOL + H_2_O	30.36^a^^b^^c^	32.91^a^^b^^c^	35.99^a^^b^^c^	5.63^a^^b^^c^	−0.3932^a^^b^^c^	33.96^a^^b^^c^	35.01^a^^b^^c^	36.01^a^^b^^c^	2.05	−0.0299	31.52^a^^b^^c^	34.82^a^^b^^c^	36.49^a^^b^^c^	4.97^a^^b^^c^	0.058
COOL + VEST + H_2_O	29.92^a^^b^^c^	31.98^a^^b^^c^^d^	34.42^a^^b^^c^^d^	4.5^a^^b^^c^^d^	−0.0814^d^	31.57^a^^b^^c^^d^	32.79^a^^b^^c^^d^	34.08^a^^b^^c^^d^	2.51^c^	0.0163	30.52^a^^b^^c^	32.74^a^^b^^c^^d^	34.9^a^^b^^c^^d^	4.38^a^^b^^c^	0.017

^a^ significantly different from SUN condition.

^b^ significantly different from SUN + H_2_O condition.

^c^ significantly different from SHADE condition. Min, minimum value; Max, maximum value; diff, mean difference pre-post activity.

^d^ significantly different from [COOL + H_2_O] condition. C**·**mins^−1^, Celsius per min.

**Figure 5:**
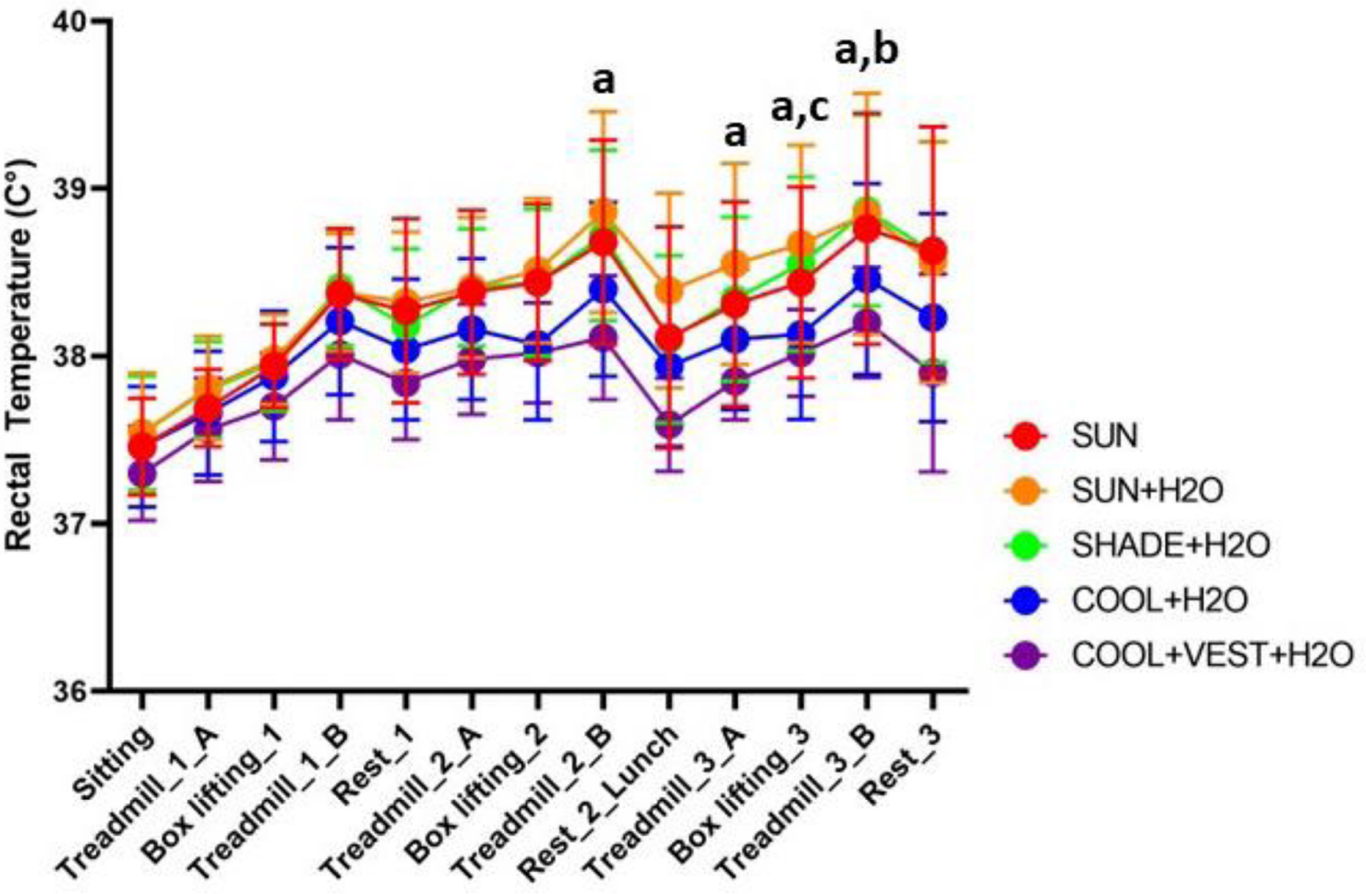
Rectal temperature responses during experimental trials. ^a^significant difference between [COOL + VEST + H_2_O] and [SUN + H_2_O]; ^b^significant difference between [COOL + H_2_O] and [SUN + H_2_O]; ^c^significant difference between [SHADE + H_2_O] and [SUN + H_2_O]. Trec, rectal temperature; C, degrees Celsius.

For cycle comparisons in box lifting activity, only the third cycle demonstrated any significant differences in Trec, where Trec was 0.45°C and 0.50°C greater in the [SUN + H_2_O] condition compared to the [COOL + H_2_O] (p = 0.014, ES = 1.459, 95% C.I. = 0.533, 2.383, *large effect*) and [COOL + VEST + H_2_O] (p = 0.005, ES = 1.611, 95% C.I = 0.677, 2.545, *large effect*) conditions, respectively (Figure 5, Table 2). For cycle comparisons for the treadmill activities, significant differences between conditions were observed during Treadmill task 2B, 3A and 3B (Figure 5). Significant differences in task 2B and 3A were between [SUN + H_2_O] and the [SUN + VEST + H_2_O] trials. Of note, during Treadmill task 3B, the [SUN + H_2_O] condition had a 0.38°C higher Trec than the [COOL + VEST + H_2_O] condition (p = 0.015, ES = 1.324, 95% C.I. = 0.484, 2.164, *large effect*), and the [SHADE + H_2_O] condition had a 0.36°C greater Trec than the [COOL + VEST + H_2_O] condition (p = 0.021, ES = 1.229, 95% C.I. = 0.426, 2.032, *large effect*).

### Skin temperature

Overall trial level comparisons reveal that Tsk was significant lower in [COOL + VEST + H_2_O] compared to [SUN] (*p* < 0.001, ES = 7.952, 95% C.I = 6.197, 9.707, *large effect*), [SUN + H_2_O] (*p* < 0.001, ES = 7.996, 95% C.I = 6.243, 9.749, *large effect*), [SHADE + H_2_O] (*p* < 0.001, ES = 7.05, 95% C.I = 5.45, 8.642, *large effect*), and [COOL + H_2_O] (*p* < 0.001, ES = 3.001, 95% C.I = 2.001, 4.002, *large effect*). Tsk was also significantly lower in [COOL + H_2_O] compared to [SUN] (*p* < 0.001, ES = 4.951, 95% C.I = 3.678, 6.223, *large effect*), [SUN + H_2_O] (*p* < 0.001, ES = 4.995, 95% C.I = 3.729, 6.261, *large effect*), and [SHADE + H_2_O] (*p* < 0.001, ES = 4.048, 95% C.I = 2.917, 5.18, *large effect*). Average skin temperature during treadmill activities were 36.92°C, 37.04°C, 36.59°C, 34.82°C, and 32.74°C during the [SUN], [SUN + H_2_O], [SHADE + H_2_O], [COOL + H_2_O], and [COOL + VEST + H_2_O], conditions, respectively (Table 2). Average skin temperature was significantly lower during the treadmill and box lifting activities across all cycles in the [COOL + VEST + H_2_O] compared to [SUN](treadmill: *p* < 0.001, ES = 6.127, *large effect*, 95% CI = 5.069–7.186, box lifting: *p* < 0.001, ES = 6.229, *large effect*, 95% CI = 5.164–7.295), [SUN + H_2_O] (treadmill: *p* < 0.001, ES = 6.245, *large effect*, 95% CI = 5.192–7.298, box lifting: *p* < 0.001, ES = 6.291, *large effect*, 95% CI = 5.235–7.347), [SHADE + H_2_O] (treadmill: *p* < 0.001, ES = 5.602, *large effect*, 95% CI = 4.593–6.610, box lifting: *p* < 0.001, ES = 2.441, *large effect*, 95% CI = 1.596–3.286), and [COOL + H_2_O] (treadmill: *p* < 0.001, ES = 3.025, *large effect*, 95% CI = 2.158–3.893, box lifting: *p* < 0.001, ES = 3.227, *large effect*, 95% CI = 2.351–4.103). While comparing cycle by cycle, there were significant differences in skin temperature between [SUN] compared to the [COOL + H_2_O] and [COOL + VEST + H_2_O] conditions during almost all box lifting and treadmill tasks (*p* < 0.05). The largest effect size was observed when comparing the [SUN] condition compared to [COOL + VEST + H_2_O] during box lifting cycle 3 (MD = 5, *p* < 0.001, ES = 5.106, 95% C.I. 4.096, 6.116) However, Tsk in the [SUN] was only significantly different from [SHADE + H_2_O] during Treadmill 1A (*p* = 0.023, ES = 1.213, 95% C.I. = 0.402, 2.024, large effect).

### Core to skin gradient

When comparing each box lifting cycle, the core to skin gradient was larger in [COOL + VEST + H_2_O] compared to [SUN], [SHADE + H_2_O], and [SHADE + H_2_O] across all three cycles (Supplementary Table S2, Figure S1) In cycles 2 and 3, the core to skin gradient was larger in [COOL + H_2_O] compared to [SUN], [SHADE + H_2_O], and [SHADE + H_2_O]. The greatest difference was between [COOL + VEST + H_2_O] and [SUN + H_2_O] with an average difference of 0.131°C (*p* < 0.001, ES = 3.952, 95% C.I = 2.901, 5.003, *large effect*). The core to skin gradient was statistically difference in [COOL + VEST + H_2_O] compared to [COOL + H_2_O] across all three cycles.

For the treadmill activities, the core to skin gradient was larger in [COOL + VEST + H_2_O] compared to [SUN], [SHADE + H_2_O], and [SHADE + H_2_O] across all three cycles (Supplementary Table S3). The greatest difference was between [COOL + VEST + H_2_O] and [SUN] with an average difference of 0.137°C (*p* < 0.001, ES = 4.11, 95% C.I. = 3.243, 4.976, *large effect*). The core to skin gradient was larger in [COOL + VEST + H_2_O] compared to [COOL + H_2_O] across all three cycles. The core to skin gradient was also larger in [COOL + H_2_O] compared to [SUN] and [SUN + H_2_O] during treadmill 1A, 2A, 3A, and 3B (*p* < 0.05).

### Heart rate

For overall trial comparisons for HR, HR was lower in [COOL + VEST + H_2_O] compared to [SUN] (*p* < 0.001, ES = 2.429, 95% C.I = 1.471, 3.387, *large effect*), [SUN + H_2_O] (*p* < 0.001, ES = 2.001, 95% C.I = 1.082, 2.92, *large effect*), and [SHADE + H_2_O] (*p* < 0.001, ES = 2.203, 95% C.I = 1.267, 2.14, *large effect*). The greatest difference was between [COOL + VEST + H_2_O] and [SUN] with an average difference of 16 bpm. HR was also significantly lower in in [COOL + H_2_O] compared to [SUN] (*p* = 0.001, ES = 0.371, 95% C.I = 2.101, 2.556, *large effect*), [SUN + H_2_O] (*p* = 0.022, ES = 1.36, 95% C.I = 1.082, 2.92, *large effect*), and [SHADE + H_2_O] (*p* = 0.005, ES = 1.438, 95% C.I = 0.561, 2.315, *large effect*).

**Figure 6:**
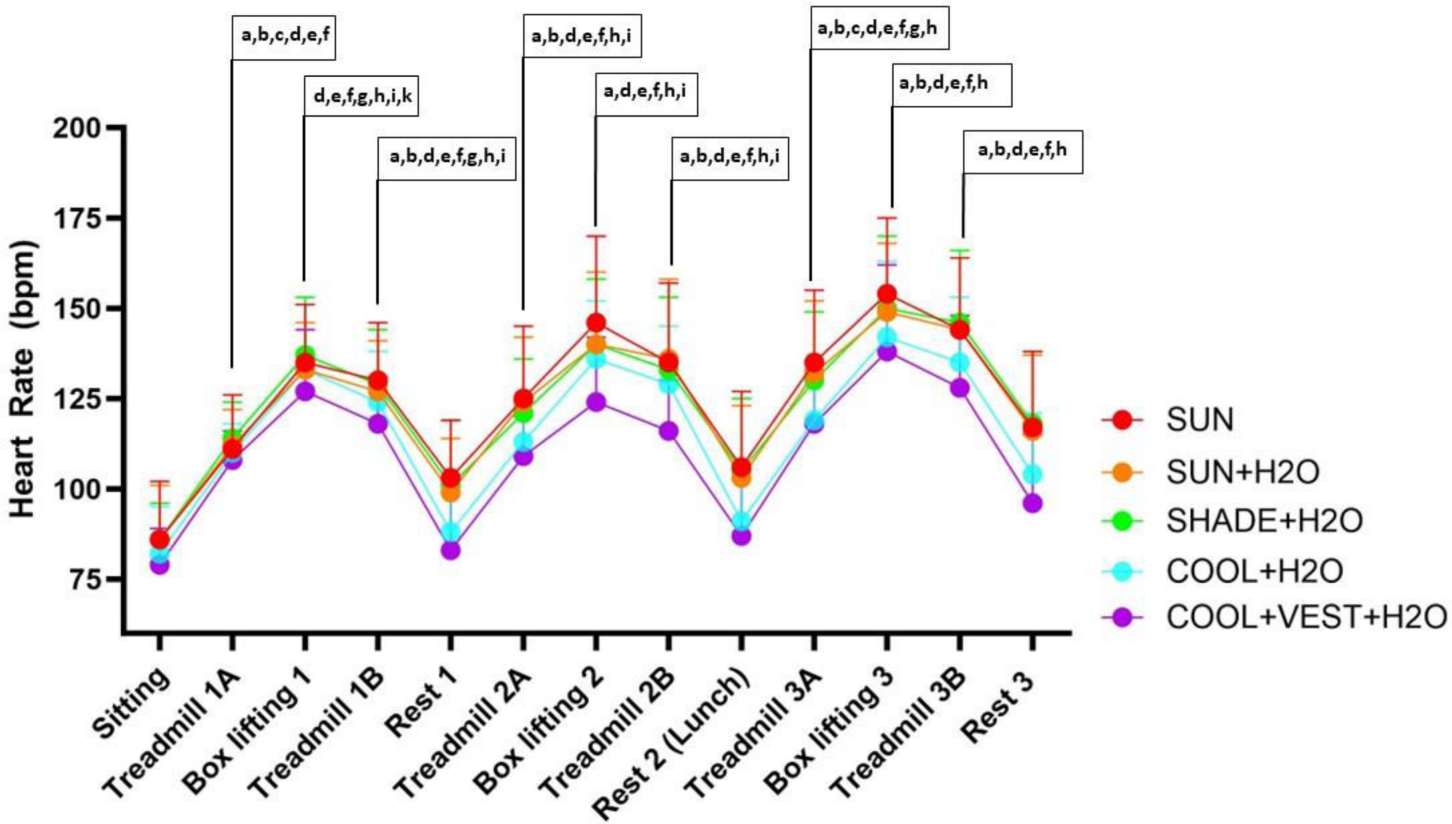
Heart rate responses during experimental trials. ^a^significant difference between [COOL + VEST + H_2_O] and [SUN + H_2_O]; ^b^significant difference between [COOL + H_2_O] and [SUN + H_2_O]; ^c^significant difference between [SHADE] and [SUN]; ^d^significant difference between [SUN] and [COOL + VEST + H_2_O]; ^e^significant difference between [SHADE + H_2_O] and [COOL + H_2_O]; ^f^significant difference between [SUN] and [COOL + VEST + H_2_O]; ^g^significant difference between [SUN] and [SUN + H_2_O]; ^h^significant difference between [COOL + H_2_O] and [SUN]; ^i^significant difference between [COOL + H_2_O] and [COOL + VEST + H_2_O]; ^k^ significant difference between [SUN + H_2_O] and [SHADE + H_2_O]. beats per minutes.

When comparing each box lifting cycle, HR was greater during the [SUN], [SUN + H_2_O], and [SHADE + H_2_O] conditions compared to the [COOL + VEST + H_2_O] condition (Figure 6). The greatest effect sizes were observed during the second box lifting activity when comparing [SUN] to [COOL + VEST + H_2_O] (*p* < 0.001, ES = 1.453, 95% C.I = 1.191, 1.716, large effect), [SUN + H_2_O] to [COOL + VEST + H_2_O] (*p* < 0.001, ES = 1.112, 95% C.I = 0.855, 1.369, large effect), and [SHADE + H_2_O] to [COOL + VEST + H_2_O] (*p* < 0.001, ES = 1.182, 95% C.I = 0.917, 1.447, large effect). Maximum HR was 169, 162, 164, 162, and 158bpm for [SUN], [SUN + H_2_O], [SHADE + H_2_O], [COOL + H_2_O], and [COOL + VEST + H_2_O], respectively.

During the treadmill activities, statistically significant differences were observed between many trials across conditions (Figure 6). The greatest effect sizes were observed when comparing the [SUN] and [SHADE + H_2_O] conditions to the [COOL + H_2_O] and [COOL + VEST + H_2_O] condition. For example, the largest ES were observed during Treadmill 2B when comparing the HR during [COOL + VEST + H_2_O] to the [SUN + H_2_O] condition (p < 0.001, ES = 1.663; 95% C.I. = 1.486, 1.84, *large effect*) and the [SHADE + H_2_O] condition to the [COOL + VEST + H_2_O] condition (*p* < 0.001, ES = 1.561, 95% C.I. = 1.379, 1.745, *large effect)*.

### Perceptual data

RPE was higher during in [SUN] compared to [COOL + VEST + H_2_O] (*p* = 0.014, ES = 1.736, 95% C.I. = 0.942, 2.53, *large effect*) following the last treadmill activity (treadmill 3B). During treadmill 3B, participants reported 14 ± 3 on the RPE scale during [SUN] compared to 11 ± 3 during [COOL + VEST + H_2_O]. There were no differences between trials at any other time point (Supplementary Table S5).

For thermal sensation, participants reported a higher thermal sensation during [SUN] and [SUN + H_2_O] compared to [COOL + VEST + H_2_O] following treadmill activity 1B, rest block 1, box lifting cycle 2, rest block 2, and box lifting cycle 3 (Supplementary Table S6). During the [COOL + H_2_O] condition compared to the [SUN] condition, participants reported lower thermal sensation following rest block 1 (*p* < 0.001, ES = 2.039, 95% C.I. = 1.25, 2.83, *large effect*) and rest block 2 (*p* < 0.001, ES = 1.977, 95%C.I. = 1.18, 2.76, *large effect*). For thermal comfort, participants reported higher thermal comfort during [COOL + VEST + H_2_O] and [COOL + H_2_O] compared to [SUN] following treadmill activity 1B, treadmill activity 2A, treadmill activity 2B, and treadmill activity 3B (*p* < 0.001, ES = 2.483, 95% C.I. = 1.69, 3.26, *large effect*; Supplementary Table S7).

Participants reported lower fatigue levels in [COOL + VEST + H_2_O] and [COOL + H_2_O] compared to [SUN] following treadmill activity 1B, treadmill activity 2A, treadmill activity 2B, and treadmill activity 3B (Supplementary Table S8). Average fatigue following treadmill activity 3B was 5 ± 3, 4 ± 2, and 3 ± 2 for [SUN], [COOL + H_2_O], and [COOL + VEST + H_2_O], respectively. Following treadmill activity 3B, fatigue was higher in [SUN] compared to [SUN + H_2_O] (*p* = 0.005, ES = 1.911, 95%C.I. = 1.08, 2.73, *large effect*).

### Box lifting

**Figure 7:**
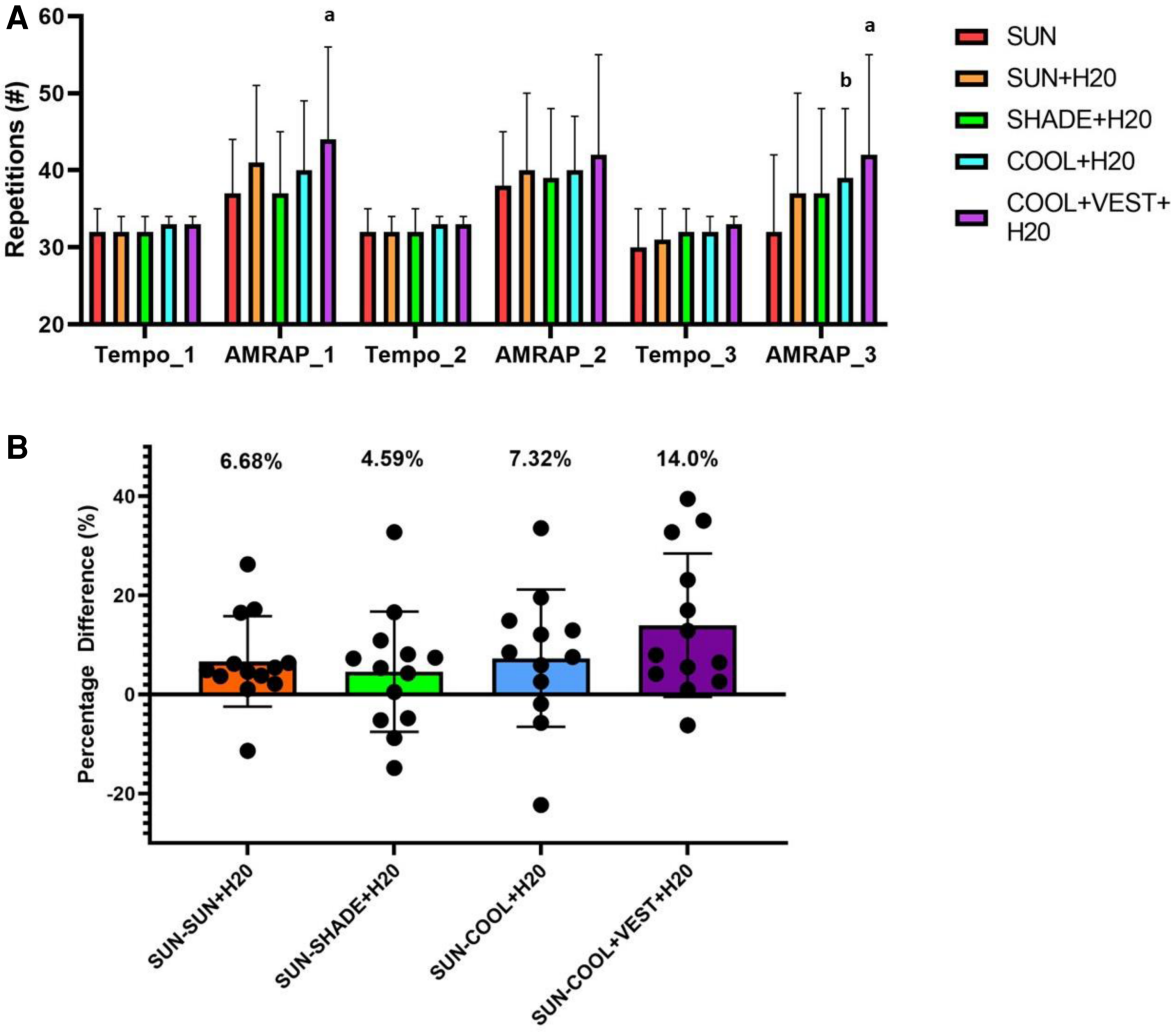
Box lifting productivity task. (**A**) Number of Box lifting Repetitions Performed Across Experimental Trials. (**B**) Percent Difference in Total Number of Boxes Lifted Compared to Control Condition [SUN]. ^a^significant difference between [COOL + VEST + H_2_O] and [SUN]; ^b^significant difference between [COOL + H_2_O] and [SUN]. #, number; TEMPO, paced box lifting following a metronome; AMRAP, as many rounds as possible.

Although not statistically significant (*p* = 0.097), there was a 6.68 ± 9.11%, 4.59 ± 12.12%, 7.32 ± 13.84%, and 14 ± 14.46% increase in total number of boxes lifted across all three cycles during the [SUN + H_2_O], [SHADE + H_2_O], [COOL + H_2_O], and [COOL + VEST + H_2_O] trials, respectively compared to [SUN](Figure 7B) There were no significant differences between the total number of boxes lifting repetitions performed across all five trials (*p* = 0.302). Seven fewer box lift repetitions were completed in Cycle 1 in the [SUN] condition compared to the [COOL + VEST + H_2_O] condition (*p* = 0.033, ES = −1.14, 95% C.I. = −1.957, −0.324, *large effect,* Figure 7A). Similarly, seven fewer repetitions were also completed in the [SUN] condition in cycle 3 compared to the [COOL + H_2_O] condition (*p* = 0.023, ES = −1.192, 95% C.I = −2.01, −0.375, large effect) and 10 fewer repetitions compared to the [COOL + VEST + H_2_O] condition (*p* = 0.07, ES = −0.968, 95% C.I = −1.789, −0.147, *large effect*).

### Time to exhaustion

**Figure 8:**
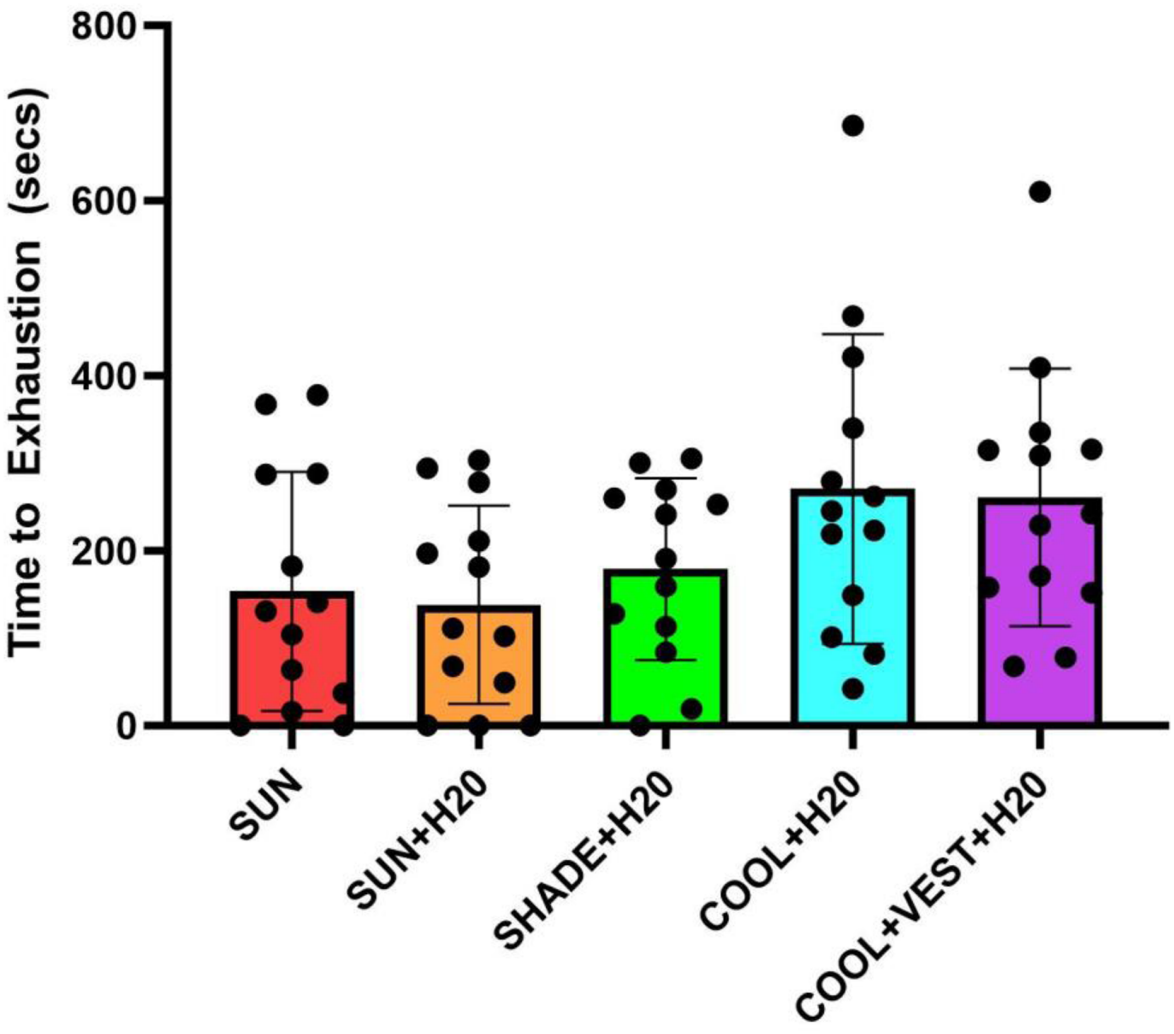
Time to exhaustion at 80% of vVO2max between experimental. ^a^significant difference between [COOL + H_2_O] and [SUN]; ^b^significant difference between [COOL + H_2_O] and [SUN + H_2_O]; ^c^significant difference between [COOL + VEST + H_2_O] and [SUN]; ^d^significant difference between [COOL + VEST + H_2_O] and [SUN + H_2_O]. vVO2peak, velocity at peak oxygen consumption; secs, seconds.

Two participants in the [SUN] condition, three participants in the [SUN + H_2_O], and one in the [SHADE] did not complete the time to exhaustion test as their core temperature values exceeded 39.75°C, which was the IRB protocol safety threshold. These six individual data points were given a “0” for the test. The data included only three total participants, where one participant could not complete three of the five time to exhaustion tests ([SUN], [SUN + H_2_O], [SHADE]), one participant could not complete two out of five tests([SUN], [SUN + H_2_O]) and one participant would could not complete one out of five tests ([SUN + H_2_O]. The time to exhaustion was 90 s and 80 s shorter in the [SUN] condition compared to the [COOL + H_2_O] (p = 0.021, ES = −1.247, 95% C.I. = −2.084, −0.409, *large effect*), and [COOL + VEST + H_2_O] (p = 0.041, ES = −1.144, 95% C.I = −1.977, −0.312, *large effect*), respectively (Figure 8). Similarly, time to exhaustion was 92 s and 82 s shorter in the [SUN + H_2_O] conditions compared to the [COOL + H_2_O] (*p* = 0.006, ES = −1.411, 95% C.I. = −2.259, −0.564, *large effect*), and [COOL + VEST + H_2_O] (*p* = 0.014, ES = −1.309, 95% C.I = −2.15, −0.468, *large effect),* respectively.

We also performed a post-hoc pseudo-sensitivity analysis of the time to exhaustion data to evaluate the influence of treating the six data points given “0” as missing data (Supplementary Figure S2). This analysis reported no statistically significant differences between time to exhaustion between trials.

### Fluid intake body mass loss, energy intake

Participants consumed an average of 2.42 ± 0.79l, 2.91 ± 0.75l, 2.58 ± 0.65l, 2.29 ± 0.88l, and 1.97 ± 0.55l in the [SUN], [SUN + H_2_O], [SHADE + H_2_O], [COOL + H_2_O], and [COOL + VEST + H_2_O] conditions, respectively. Fluid consumption differences were only statistically significant between the [SUN + H_2_O] trial and [COOL + VEST + H_2_O] trial (*p* = 0.004), where fluid consumption was higher in [SUN + H_2_O] compared to [COOL + VEST + H_2_O]. Percent body mass loss (%BML) was 1.27 ± 1.20%, 1.71 ± 2.98%, 1.20 ± 1.0%, 0.77 ± 0.90%, and 0.84 ± 0.78% for [SUN], [SUN + H_2_O], [SHADE + H_2_O], [COOL + H_2_O], AND [COOL + VEST + H_2_O], respectively. There were no significant differences in %BML or energy intake between trials. (*p* > 0.05).

## Discussion

This study investigated the effect of different heat mitigation strategies on productivity and thermoregulatory responses during simulated occupational work in a hot environment. The study used a novel experimental design that simulated a prolonged work environment and assessed the effectiveness of increased heat protection strategies that can be easily implemented in most occupational settings (i.e., fluid accessibility, shade, and body cooling). Overall trial comparisons suggested that Trec, Tsk, and HR were lower in [COOL + VEST + H_2_O] compared to [SUN], [SUN + H_2_O], and [SHADE + H_2_O]. Mean differences between minimum and maximum Trec values were lower (0.98°C) in [COOL + VEST + H_2_O] compared to [SUN] (1.42°C), [SUN + H_2_O] (1.60°C), and [SHADE + H_2_O] (1.37°C). The core to skin gradient was larger in [COOL + VEST + H_2_O] compared to [SUN], [SHADE + H_2_O], and [SHADE + H_2_O] during all box lifting and treadmill activities. HR and Tsk were lower in [COOL + H_2_O] compared to [SUN], [SUN + H_2_O], and [SHADE + H_2_O] but there were no differences in Trec. For productivity outcomes, there was no overall difference between trials in box lifting performance. However, we found that participants lifted more boxes in the [COOL + VEST + H_2_O] and [COOL + H_2_O] conditions compared to [SUN] in cycle 1 and cycle 3, respectively. The time to exhaustion was 90seconds and 80 s shorter in the [SUN] and condition compared to the [COOL + H_2_O] and [COOL + VEST + H_2_O]. For perceptual data, fatigue, thermal sensation, and RPE was lower and thermal comfort was higher in [COOL + VEST + H_2_O] and [COOL + H_2_O] compared to [SUN] throughout various time points in the trial. Therefore, the current study suggests that the addition of heat stress mitigation strategies can result in differences in physiological and perceptual parameters, however, these differences did not produce statistically significant differences in overall box lifting activity performance. Moreover, although time to exhaustion was longer in [COOL + H_2_O] and [COOL + VEST + H_2_O] compared to [SUN] and [SUN + H_2_O], this data can only be applied to working situations that require heavy physical exertion at the end of the workday to potentially overcome missed productivity goals.

For Trec, our findings were similar to previous literature examining body cooling between and during exercise bouts (29–34). Like the current study, Barr et al. (2009) examined the influence of body cooling during a 15-minute rest break between 20-minute treadmill walking bouts in firefighters. The cooling protocol consisted of a combined cooling approach using hand cooling, forearm cooling, and an ice vest. The mixed body cooling method approach was similar to the current study with both cooling towels and an ice vest to enhance cool capacity. In both studies, core temperature differences between cooling and control conditions were approximately 0.5°C (34). Differences of 0.5°C between conditions were higher than many other studies that focused on one cooling modality alone (15, 35). Tsk for [COOL + H_2_O] and [COOL + VEST + H_2_O] maintained below 34°C, which may facilitate enhanced conductive heat transfer from the core to skin, limiting the rise in Trec during activity. Moreover, the current study reported a larger core to skin gradient in [COOL + VEST + H_2_O] compared to [SUN], [SHADE + H_2_O], and [SHADE + H_2_O] during all box lifting and treadmill activities. A larger core to skin gradient facilitates enhanced heat loss as thermal energy moves down energy gradients from a higher temperature to a lower temperature. Lower Tsk values attributed to body cooling would enhance heat loss from the core to the periphery, resulting in a maintenance of a lower Trec value (36). The enhanced core to skin gradient and lower Trec, Tsk, and HR, is likely attributed to the use of multiple body cooling modalities (vest and body cooling towels) used in the current study (14). The use of multiple cooling modalities together has been shown to be an effective body cooling strategy as cool capacity is a function of body surface area covered, the temperature of the cooling modality, and the duration of cooling (14, 15, 22, 37). This cooling methodology reflects what is currently described as the “best practice” model for body cooling under heat stress (15). In the current study's rest breaks, our study cooled participants for 15–30 min, covering the head, back, chest, and thighs and replacing the cooling towels every 5 min to optimize effectiveness.

The [COOL + VEST + H_2_O] trial implemented an ice vest during physical activity with the cooling towels during rest, which has been reported to have the greatest effect on performance and physiology (21). At the end of the trial, there was a 0.72°C difference between [SUN] and [COOL + VEST + H_2_O]. Other studies, such as Selkirk et al. (2004), reported a 0.38°C reduction in core temperature after 20 min of extremity cooling alone, which differed from the best practice approach by cooling less body surface area than the current study and Barr et al. (34, 35). Although the addition of the cooling vest in the [COOL + VEST + H_2_O] did not result in statistically significant differences physiological parameters compared to the [COOL + H_2_O], [COOL + VEST + H_2_O] produced a 0.32°C reduction in core temperature compared to [COOL + H_2_O], which was the greatest difference reported compared previous studies (15, 20, 38). Therefore, worksites exposed to high heat exposure should consider providing body cooling options such as cooling vests and cooling towels during physical activity and rest to enhance body cooling potential. Like the current study, ice vest inserts should be replaced regularly to enhance cooling capacity and maintain a cool Tsk to maintain a larger core to skin gradient.

The cooling methodology performed in the study, cooling during activity, has been shown to result in a 9.3% improvement in performance tasks such as a time to exhaustion or a time trial test (15). The underlying mechanisms that contribute to the effect of cooling during activity on productivity enhancement include increased heat storage capacity, attenuated rise in core temperature, and enhanced heat loss efficiency (15, 20, 22). It has been proposed that performance outcomes in the heat are negatively impacted by critically high core temperature (39). However, it is now well documented that physically fit individuals can sustain high core temperature values with little impairment in their performance (40). Ely et al. (2009) suggested that performance in the heat relies on skin blood flow to transfer body heat from the body core to the periphery, which can be adequately maintained or enhanced by lower skin temperature values and a large core to skin gradient. Moreover, Ely et al. (2010) reported that performance during a 15-min time trial in the heat, followed a 30 min of cycling at 50% of VO_2_peak, may have been reduced by skin temperature alone, attributing to a 15%–20% reduction in time trial performance (41). Therefore, the positive effects of body cooling on productivity and its ability to increase heat storage capacity, attenuate rise in core temperature, and enhance heat loss efficiency, is likely attributed to the promotion of a large core to skin gradient as reported in the current study. Although the current study uniquely examined the influence of different heat stress mitigation strategies during a simulated work shift on productivity using an occupational simulated work activity (i.e., box lifting) tasks, the current study only reported cycle-level (cycle 1 and 3) differences in number of box lifting repetitions when heat stress mitigation strategies were added. These differences did not reflect overall trial differences in total box lifting repetitions. In other words, the addition of heat stress mitigation strategies did not statistically improve overall working productivity, but rather, reflected differences in timepoint specific data.

Given that productivity is a key performance indicator for companies alike and typically measured at the end of work shift, this is important to consider when contextualizing the results of the current study. As differences were only seen at cycle 1 ([COOL + VEST + H_2_O] vs. [SUN]) and cycle 3 ([COOL + H_2_O] and [COOL + VEST + H_2_O] vs. [SUN]), it is possible that differences were dependent of the participant's level of exhaustion, fatigue, and exposure to simulated solar radiation during the [SUN] trial (37, 38). Perceptual responses (ex: rating of perceived exertion, lower thermal sensation/comfort) have been reported to influence self-selected exercise intensity through behavioral thermoregulation, which would influence the current study's productivity outcomes (43–45). Participants may have altered their pace during the AMRAP protocol based on their perceived exertion or thermal comfort and may not have been a different influence of physiological measures *per se* (43–45)*.* In the current study, we found that fatigue, RPE, and thermal sensation were lower in various time points [COOL + H_2_O] and [COOL + VEST + H_2_O] compared to [SUN]. However, there were only perceptual response differences in thermal sensation prior to performing each box lifting activity (following rest blocks, pre treadmill activities A). In all trials, participants were required to perform cycle 2 with only a 15 min rest period between cycle 1 and 2 (i.e., limited rest), whereas participants were able to rest for 30 min with a lunch break before the start of cycle 3. The rest periods in the [SUN] condition were also performed with simulated solar radiation exposure with no exposure of simulated solar radiation during any point of the cooling trials. Ioannou et al. (2021) reported that during occupational field research studies in the heat, working in the sun exacerbated physiological heat strain and cognitive function, even when the level of heat stress is thought to be the same as the same conditions with no sun exposure (42). For example, workers exposed to solar radiation during their work shift have a 45% and 67% reduction in cognitive attention and vigilance compared to the same working conditions without solar radiation (i.e., shade) (42). Future studies must examine the influence of heat stress mitigation strategies during sun exposure or simulated solar radiation to identify whether differences exist in the presence of increased thermal strain. Therefore, the impact of heat stress mitigation strategies such as body cooling may improve occupational productivity during times of high thermal strain (ex: high core temperature, high heart rate), however, more research must further explore this relationship.

As previously noted, there were no differences in physiological responses or productivity outcomes when fluid was restricted during physical activity in the [SUN] compared to [SUN + H_2_O] trials. It is possible that a 0.49l greater fluid intake in the [SUN + H_2_O] did not produce clinically meaningful changes to promote differences in physiological responses and productivity outcomes in [SUN] vs. [SUN + H_2_O]. Although fluid was restricted during physical activity within the [SUN] trial, participants were permitted to consume fluids *ad libitum* during rest breaks, which may have resulted in the absence of differences between conditions. The ability for participants to drink *ad libitum* during rest breaks did not result in greater %BML (1.27 ± 1.20%) compared to other trials ([SUN + H_2_O] = 1.71 ± 2.98%, [SHADE + H_2_O] = 1.20 ± 1.0%, [COOL + H_2_O] = 0.77 ± 0.90%, and COOL + VEST + H_2_O] = 0.84 ± 0.78%). A recent study by Pryor et al. (2023) examined two divergent water consumption patterns (237 ml every 20 min for 2 h or 500 ml every 40 min for 2 h) during exercise in the heat and reported that there were no differences in hydration biomarkers between trials. In the current study, participants may have consumed larger boluses of fluids during rest breaks rather than smaller boluses throughout the trial, producing similar differences in BML. Moreover, on average, %BML across all trials did not exceed 2%, and there is strong evidence to suggest that physiological and performance decrements occur when BML is greater or equal to 2% (47–49). These findings are encouraging for workers who may have limited availability of fluids during work, as drinking *ad libitum* during rest breaks only did not influence physiology or productivity.

To our knowledge, the current study is the first to examine the effectiveness of heat mitigation strategies (hydration, shade, body cooling) during simulated occupational work. The study utilized solar radiation lamps to mimic the conditions that workers may be exposed to during work in the heat. Although our study has several strengths, there are several limitations and considerations to address. For the time to exhaustion test, there were six incidences where participants could not perform the test due to IRB safety concerns (Trec over 39.75°C) and the data points were included in the analysis with a “0” time to exhaustion score. Ecologically, we acknowledge that many physically active individuals tolerate core temperatures that exceed 39.75°C. However, these data points were not excluded to eliminate bias and the risk of reducing the true effect of the condition. The exclusion of the data (Supplementary Figure S2) reported no statistically significant differences (*p* > 0.05) despite relatively large effect sizes. Second, the ice vests used in the [COOL + VEST] were replaced every 30 min to ensure that aggressive cooling was maintained. In many work site conditions, it is not feasible to continuously replace the ice inserts frequently, which would limit the optimal effect of the ice vest. Moreover, we did not measure the temperature of the ice pack every 30 min to evaluate the cooling capacity of the ice pack inserts in the vests. Interestingly, the current study did not find any differences in cooling rates despite differences in Trec (Table 2). As body cooling capacity is a function of duration of cooling, it is possible that the duration of body cooling (two 15 min blocks, one 30 min block) was responsible for the lack of statistically significant differences. There were also only differences in Trec across 4 of 13 time points assessed, which could account for the absence of differences in cooling rates. However, more research is needed to determine the mechanism that resulted in these findings. An additional limitation was the inclusion of five experimental trials, which increases the likelihood of a training effect. To mitigate the influence of multiple trials, each trial was randomized to the order in which each participant performed the trials. We also did not randomize the order in which the participants performed the TEMPO and AMRAP protocols. Our study was performed in healthy, physically active young men, representing only a small portion of laborers working in hot conditions. Women make up approximately 49% of the U.S workforce. Women were not included in the current study based on the funding available and the sponsor's (MISSION, LLC) decision. Many laborers also have individual characteristics (i.e., obesity, hypertension, diabetes) that may influence their ability to thermoregulate in the heat. Studies on the impact of body cooling in these vulnerable working populations must be performed to enhance the generalizability of our findings.

## Conclusion

The addition of heat stress mitigation strategies (body cooling, hydration, removal of simulated radiant heat; [COOL + H_2_O] and [COOL + VEST + H_2_O]) during simulated occupational work produced the greatest reductions in Trec, Tsk, and HR. These differences may have been attributed to a larger core to skin temperature gradient during [COOL + H_2_O] and [COOL + VEST + H_2_O] or a reduction in fatigue, RPE or thermal sensation. Although participants were able to lift more boxes in the [COOL + VEST + H_2_O] and [COOL + H_2_O] conditions compared to [SUN] in cycle 1 and cycle 3, respectively, these differences did not improve overall work productivity. Given the benefits of the additional heat stress mitigation strategies on physiological parameters and productivity during cycle 1 and cycle 3, this intervention can be considered on an individual basis as it is does not provide any additional harm.

## Data Availability

The raw data supporting the conclusions of this article will be made available by the authors, without undue reservation.
