# Supplementary material for: The effect of heat mitigation strategies on thermoregulation and productivity during simulated occupational work in the heat in physically active young men
Source: Front Sports Act Living. 2024 Jan 11;5:1274141. doi: 10.3389/fspor.2023.1274141 (PMC10808760; doi:10.3389/fspor.2023.1274141)
Supplement: Supplementary file 1 [file Datasheet1.docx]

1. Borg MA, Xiang J, Anikeeva O, Pisaniello D, Hansen A, Zander K, et al. Occupational heat stress and economic

burden: a review of global evidence. Environ Res. (2021) 195:110781. doi: 10.1016/j.

envres.2021.110781

2. Morrissey MC, Brewer GJ, Williams WJ, Quinn T, Casa DJ. Impact of occupational heat stress

on worker productivity and economic cost. Am J Ind Med. (2021) 64(12):981–8.

doi: 10.1002/ajim.23297

3. Day E, Fankhauser S, Kingsmill N, Costa H, Mavrogianni A. . Upholding labour productivity under

climate change: an assessment of adaptation options. Clim Policy. (2019) 19:367–85.

doi: 10.1080/14693062.2018.1517640

4. Flouris AD, Dinas PC, Ioannou LG, Nybo L, Havenith G, Kenny GP, et al. Workers’ health and productivity under

occupational heat strain: a systematic review and meta-analysis. Lancet Planet Health.

(2018) 2:e521–31. doi: 10.1016/S2542-5196(18)30237-7

5. Ioannou LG, Mantzios K, Tsoutsoubi L, Panagiotaki Z, Kapnia AK, Ciuha U, et al.. Effect of a simulated heat wave on

physiological strain and labour productivity. Int J Environ Res Public Health. (2021)

18:3011. doi: 10.3390/ijerph18063011

6. Gun RT, Budd GM. Effects of thermal, personal and behavioural factors on the

physiological strain, thermal comfort and productivity of Australian shearers in hot

weather. Ergonomics. (1995) 38:1368–84. doi: 10.1080/00140139508925195

7. Lee S-W, Lee K, Lim B. Effects of climate change-related heat stress on labor

productivity in South Korea. Int J Biometeorol. (2018) 62(12):2119–29. doi: 10.1007/

s00484-018-1611-6

8. Morabito M, Messeri A, Crisci A, Bao J, Ma R, Orlandini S, et al. Heat-related productivity loss: benefits

derived by working in the shade or work-time shifting. Int J Product Perform

Manag. (2020) 70:507–25. doi: 10.1108/IJPPM-10-2019-0500

9. Orlov A, Sillmann J, Aaheim A, Aunan K, de Bruin K. Economic losses of heat-induced reductions

in outdoor worker productivity: a case study of Europe. EconDisCliCha. (2019)

3:191–211. doi: 10.1007/s41885-019-00044-0

10. Foster J, Smallcombe J, Hodder S, Jay O, Flouris AD, Nybo A, et al. . An advanced empirical model for

quantifying the impact of heat and climate change on human physical work

capacity. Int J Biometeorol. (2021):65(7): 1215-1229. doi: 10.1007/s00484-021-02105-0.¶

11. Miller V, Bates G, Schneider JD, Thomsen J. . Self-pacing as a protective mechanism

against the effects of heat stress. Ann Occup Hyg. (2011) 55:548–55. doi: 10.1093/

annhyg/mer012

12. Miller VS, Bates GP. The thermal work limit is a simple reliable heat index for

the protection of workers in thermally stressful environments. Ann Occup Hyg. (2007)

51:553–61. doi: 10.1093/annhyg/mem035.

13. Data and Statistics|U.S. Department of Labor. Available at: https://www.dol.gov/

agencies/wb/data (Accessed November 10, 2020)

14. DeMartini JK, Ranalli GF, Casa DJ, Lopez RM, Ganio MS, Stearns RL, et al. Comparison of body cooling

methods on physiological and perceptual measures of mildly hyperthermic

athletes. J Strength Cond Res. (2011) 25:20 65–74. doi: 10.1519/JSC.0b013e3182259b1d.15. Bongers CCWG, Hopman MTE, Eijsvogels TMH. Cooling interventions for

athletes: an overview of effectiveness, physiological mechanisms, and practical

considerations. Temperature (Austin). (2017) 4:60–78. doi: 10.1080/23328940.2016.

1277003

16. Brearley M. Cooling methods to prevent heat-related illness in the workplace.

Workplace Health Saf. (2016) 64:80–80. doi: 10.1177/216507991561335317.

17. Chicas R, Xiuhtecutli N, Dickman NE, Scammell ML, Steenland K, Hertzberg VS, et al. Cooling intervention studies among

outdoor occupational groups: a review of the literature. Am J Ind Med. (2020)

63:988–1007. doi: 10.1002/ajim.23175

18. Zhao M, Gao C, Li J, Wang F.. Effects of two cooling garments on post-exercise

thermal comfort of female subjects in the heat. Fibers Polym. (2015) 16:1403–9.

doi: 10.1007/s12221-015-1403-0

19. Yang H, Cao B, Ju Y, Zhu Y.. The effects of local cooling at different torso parts in

improving body thermal comfort in hot indoor environments. Energy Build. (2019)

198:528–41. doi: 10.1016/j.enbuild.2019.06.004

20. Stevens CJ, Taylor L, Dascombe BJ. Cooling during exercise: an overlooked

strategy for enhancing endurance performance in the heat. Sports Med. (2017)

47:829–41. doi: 10.1007/s40279-016-0625-7

21. Tyler CJ, Sunderland C, Cheung SS. The effect of cooling prior to and during

exercise on exercise performance and capacity in the heat: a meta-analysis. Br

J Sports Med. (2015) 49:7–13. doi: 10.1136/bjsports-2012-091739

22. Douzi W, Dugué B, Vinches L, Al Sayed C, Halle S, Bosquet L, et al. Cooling during exercise enhances

performances, but the cooled body areas matter: a systematic review with metaanalyses.

Scand J Med Sci Sports. (2019) 29:1660–76. doi: 10.1111/sms.13521

23. Ramanathan NL. A new weighting system for mean surface temperature of the

human body. J Appl Physiol. (1964) 19:531–3. doi: 10.1152/jappl.1964.19.3.531

24. Imboden MT, Kaminsky LA, Peterman JE, Hutzler HL, Whaley MH, Fleenor BS, et al. Cardiorespiratory fitness

normalized to fat-free mass and mortality risk. Med Sci Sports Exerc. (2020)

52:1532–7. doi: 10.1249/MSS.0000000000002289

25. Butts CL, Smith CR, Ganio MS, McDermott BP.. Physiological and perceptual effects of a

cooling garment during simulated industrial work in the heat. Appl Ergon. (2017)

59:442–8. doi: 10.1016/j.apergo.2016.10.001

26. R Core Team. R: A language and environment for statistical computing. Vienna,

Austria: R Foundation for Statistical Computing (2013). Available at: http://www.Rproject

org/

27. Bates D, Mächler M, Bolker B, Walker S.. Fitting Linear Mixed-Effects Models Using

lme4. arXiv [preprint]. (2014). Available at: https://arxiv.org/abs/14065823

28. Lenth R, Bolker B, Buerkner P, Giné-Vázquez I, Herve M, Jun M, et al.et al.. Package ‘Emmeans’. (2019).

29. Cleary MA, Toy MG, Lopez RM. Thermoregulatory, cardiovascular, and

perceptual responses to intermittent cooling during exercise in a hot, humid

outdoor environment. J Strength Cond Res. (2014) 28:792–806. doi: 10.1519/JSC.

0b013e3182a20f57

30. Schranner D, Scherer L, Lynch GP, Korder S, Brotherhood JR, Pluim BM, et al. In-play cooling interventions for

simulated match-play tennis in hot/humid conditions. Med Sci Sports Exerc. (2017)

49:991–8. doi: 10.1249/MSS.0000000000001183

31. Lynch GP, Périard JD, Pluim BM, Brotherhood JR, Jay O. . Optimal cooling strategies for players in

Australian tennis open conditions. J Sci Med Sport. (2018) 21:232–7. doi: 10.1016/j.

jsams.2017.05.017

32. Chalmers S, Siegler J, Lovell R, Lynch G, Gregson W, Marshall P, et al. Brief in-play cooling breaks reduce thermal

strain during football in hot conditions. J Sci Med Sport. (2019) 22:912–7. doi: 10.1016/

j.jsams.2019.04.009

33. Naito T, Sagayama H, Akazawa N, Haramura M, Tasaki M, Takahashi H.. Ice slurry ingestion during break times

attenuates the increase of core temperature in a simulation of physical demand of

match-play tennis in the heat. Temperature (Austin). (2018) 5:371–9. doi: 10.1080/

23328940.2018.1475989

34. Barr D, Gregson W, Sutton L, Reilly T.. A practical cooling strategy for reducing the

physiological strain associated with firefighting activity in the heat. Ergonomics. (2009)

52:413–20. doi: 10.1080/00140130802707675

35. Selkirk GA, McLellan TM, Wong J. Active versus passive cooling during work in

warm environments while wearing firefighting protective clothing. J Occup Environ

Hyg. (2004) 1:521–31. doi: 10.1080/15459620490475216

36. Institute of Medicine (US) Committee on Military Nutrition Research.

Nutritional needs in hot environments: applications for military personnel in field

operations. In: Marriott BM, editors. Physiological Responses to Exercise in the Heat.

Washington, DC: National Academies Press (US) (1993). 55-75 .

¶

37. Casa DJ, McDermott BP, Lee EC, Yeargin SW, Armstrong LE, Maresh CM. Cold water immersion: the gold standard

for exertional heatstroke treatment. Exerc Sport Sci Rev. (2007) 35:141–9. doi: 10.1097/

jes.0b013e3180a02bec

38. Stevens CJ, Bennett KJM, Sculley DV, Callister R, Taylor L, Dascombe BJ. . A comparison of mixed-method

cooling interventions on preloaded running performance in the heat. J Strength

Cond Res. (2017) 31:620–9. doi: 10.1519/JSC.0000000000001532

39. González-Alonso J, Teller C, Andersen SL, Jensen FB, Hyldig T, Nielsen B. . Influence of body temperature

on the development of fatigue during prolonged exercise in the heat. J Appl Physiol

(1985). (1999) 86:1032–9. doi: 10.1152/jappl.1999.86.3.1032

40. Ely BR, Ely MR, Cheuvront SN, Kenefick RW, Degroot DW, Montain SJ. . Evidence against a 40 degrees C core

temperature threshold for fatigue in humans. J Appl Physiol (1985). (2009) 107

(5):1519–25. doi: 10.1152/japplphysiol.00577.2009

41. Ely BR, Cheuvront SN, Kenefick RW, Sawka MN. Aerobic performance is degraded,

despite modest hyperthermia, in hot environments. Med Sci Sports Exerc. (2010)42:135–41. doi: 10.1249/MSS.0b013e3181adb9fb

42. Ioannou LG, Tsoutsoubi L, Mantzios K, Gkikas G, Piil JF, Dinas PC, et al. The impacts of sun exposure on

worker physiology and cognition: multi-country evidence and interventions. Int

J Environ Res Public Health. (2021) 18:7698. doi: 10.3390/ijerph18147698

43. Périard JD, Eijsvogels TMH, Daanen HAM. Exercise under heat stress:

thermoregulation, hydration, performance implications, and mitigation strategies.

Physiol Rev. (2021) 101(4):1873–979. doi: 10.1152/physrev.00038.2020

¶

44. James CA, Hayes M, Willmott AGB, Gibson OR, Flouris AD, Schlader ZJ, et al. Defining the determinants of

endurance running performance in the heat. Temperature (Austin). (2017)

4:314–29. doi: 10.1080/23328940.2017.1333189

45. Schlader ZJ, Simmons SE, Stannard SR, Mündel T. The independent roles of

temperature and thermal perception in the control of human thermoregulatory

behavior. Physiol Behav. (2011) 103:217–24. doi: 10.1016/j.physbeh.2011.02.002

46. Schlader ZJ, Perry BG, Jusoh MRC, Hodges LD, Stannard SR, Mündel T. Human temperature regulation when

given the opportunity to behave. Eur J Appl Physiol. (2013) 113:1291–301. doi: 10.

1007/s00421-012-2544-0

47. Cheuvront SN, Kenefick RW. Dehydration: physiology, assessment, and

performance effects. Compr Physiol. (2014) 4:257–85. doi: 10.1002/cphy.c130017

48. Cheuvront SN, Kenefick RW, Montain SJ, Sawka MN. Mechanisms of aerobic

performance impairment with heat stress and dehydration. J Appl Physiol. (2010)

109:1989–95. doi: 10.1152/japplphysiol.00367.2010

49. Goulet EDB. Effect of exercise-induced dehydration on endurance

performance: evaluating the impact of exercise protocols on outcomes using a

meta-analytic procedure. Br J Sports Med. (2013) 47:679–86. doi: 10.1136/

bjsports-2012-090958
